# Inhibition of Retinoblastoma Cell Growth by Boswellic Acid Through Activation of the Suppressing Nuclear Factor—κB Activation

**DOI:** 10.3390/medicina61030480

**Published:** 2025-03-10

**Authors:** Semih Doğan, Mehmet Cudi Tuncer, İlhan Özdemir

**Affiliations:** 1 Department of Ophthalmology, Faculty of Medicine, Beykent University, İstanbul 34398, Turkey; drsemih@hotmail.com; 2 Department of Anatomy, Faculty of Medicine, Dicle University, Diyarbakır 21280, Turkey; 3 Department of Gynecology and Obstetrics, Faculty of Medicine, Atatürk University, Erzurum 25240, Turkey; ilhanozdemir25@yandex.com

**Keywords:** retinoblastoma, *NF-κB* gene, boswellic acid, oxidative stress

## Abstract

*Background and Objectives:* Despite the development of treatment methods and the emergence of alternative new approaches in recent years, the visual prognosis of retinoblastoma contains deficiencies and this situation increases the need for the development of new treatment approaches. The cytotoxic and apoptosis-inducing effects of the combination of boswellic acid (BA), which has been determined to have significant potential in preclinical and clinical studies of various diseases, and Cisplatin (Cis), a potent chemotherapy agent, were investigated on the human retinoblastoma cell line (Y79). *Materials and Methods:* The cytotoxic effect of BA and Cis on Y79 cells was determined by the water soluble tetrazolium-1 (WST-1) test, the apoptotic rate of the cells was determined by annexin V staining, and the gene expressions of *Protein53 (p53)*, *Caspase-3* and *Nuclear factor kappa B (NF-κB)*, which play an important role in apoptosis, were determined by RT-qPCR analysis. Interleukin 1-beta (IL1-β), tumor necrosis factor-α (TNF-α) and interferon γ (IFN-γ) levels were analyzed in cell lysates obtained from the experimental groups. *Results:* The combination of BA and Cis selectively inhibited the growth of Y79 cells and modulated *NF-κB* signaling, potentially through post-translational regulatory mechanisms. Moreover, it induced apoptosis by increasing *p53* and *Caspase-3* expressions, confirming its pro-apoptotic effects. Additionally, the combination treatment was associated with a reduction in inflammatory cytokine levels (TNF-α, IL1-β), suggesting a potential regulatory effect on inflammation-related pathways rather than direct inhibition of *NF-κB* activation. *Conclusions:* These findings suggest that BA combined with Cis inhibits Y79 retinoblastoma cell growth by inducing apoptosis and modulating *NF-κB* signaling. While *NF-κB* mRNA levels increased, reduced inflammatory cytokines and enhanced apoptosis suggest potential post-translational regulation. Further studies are needed to confirm *NF-κB* protein-level effects and in vivo efficacy.

## 1. Introduction

Retinoblastoma is the most common intraocular tumor of childhood. It develops from the inner layer of the eye (retina), where the optic nerves are located, and usually grows into the eyeball. Patients are diagnosed on average around the age of two. Approximately 80% of children are diagnosed before the age of three. As can be understood, retinoblastoma is usually a disease of young children. However, there is a risk of tumor development up to the age of six or seven [1,2]. The main goal of retinoblastoma treatment is to protect the patient’s life and then to save the eye and visual function. The treatment methods used today are approaching this goal, but they bring with them significant side effects and heavy costs to the patient and the healthcare system. Therefore, alternative treatment searches continue.

In recent years, natural products have been shown to have effective results against retinoblastoma, either in combination with standard drugs or alone [3,4]. Current treatment options for retinoblastoma are radiotherapy, chemotherapy, thermotherapy and brachytherapy [5]. Many complications arise due to traditional treatments. Secondary cancers caused by traditional treatments also occur in younger people [6]. The importance of plant-derived natural products in cancer treatment cannot be ignored. Many drugs used in current treatment such as Paclitaxel, camptothecin, etoposide, vinblastine and vincristine are plant-derived natural products [7]. Effective results of the combination of plant-derived agents and Cisplatin have been reported in many cancer treatments. Boswellic acid, which contains many triterpenes, has been shown to have apoptotic, cytostatic and antiproliferative activities against different cancers both in vivo and in vitro [8,9]. It has also been reported that BA can increase apoptosis together with chemotherapeutic agents. It has been reported to prevent invasive cancers as a result of inhibition of *NF-κB*-regulated gene expression [10]. Moreover, it prevents mitochondrial apoptosis by activating activated *Caspase-8* to *Caspase-3* [11].

*NF-κB* is an important transcription factor in cells and has an important role in the immune system through various cellular responses [12]. Research confirms that inflammation is closely related to the formation and development of cancers, and *NF-κB*, a key molecule linking chronic inflammation to cancer [13], is an important subject of antitumor studies.

In this study, we investigate the impact of BA and its combination with Cis on Y79 retinoblastoma cells, focusing on cell viability, apoptosis induction, *NF-κB* modulation, and inflammatory cytokine levels. Our findings aim to provide insights into the therapeutic potential of BA in retinoblastoma management and contribute to the development of alternative treatment strategies.

## 2. Material and Methods

### 2.1. Cell Culture

Human retinoblastoma cell line Y79 was obtained from American Type Culture Collection (ATCC^®^ HTB-18^TM^, Manassas, VA, USA). Y79 was used to determine the effects of boswellic acid and Cisplatin on inflammation, oxidative stress and apoptosis in retinoblastoma cancer. The Y79 cell line was commercially obtained (ATCC) and the medium used for the propagation of its cells was prepared to contain 10% (*v*/*v*) Fetal bovine serum (Gibco, ThermoFisher, Waltham, MA, USA), 100 µg/mL streptomycin (Invitrogen, ThermoFisher, USA) and RPMI-1640 (Sigma-Aldrich, Merck, St. Louis, MO, USA). After the applications to the cells, all incubations were carried out in a sterile environment with 5% CO_2_ and 37 °C incubator that maintained the balance. 

### 2.2. Determination of Boswellic Acid and Cisplatin Concentration

Acetyl-11-Keto-Beta-Boswellic Acid (Santa Cruz, CA, USA) and CISPLATIN DBL (Hospira Australia Pty Ltd., Mulgrave-Victoria, Australia) were obtained commercially. Optimization experiments were performed for BA and Cis concentrations of 0.5 μM, 1 μM, 2 μM, 5 μM, 10 μM, 25 μM, 50 μM, 75 μM and 100 μM and the lethal dose of BA and Cis on human retinoblastoma cancer cells were determined. A total of 5 mg of BA in the stock was dissolved in DMSO and then divided into aliquots by adding distilled water. To determine the concentration, WST-1 assay was performed to determine the lethal dose of BA and Cis on human retinoblastoma cancer cells. WST-1 test was performed to determine half maximal inhibitory concentration (IC_50_) doses.

### 2.3. WST-1 Assay

In order to determine cell proliferation, 0.5 μM, 1 μM, 2 μM, 5 μM, 10 μM, 25 μM, 50 μM, 75 μM and 100 μM BA and Cis concentrations were added to Y79 cancer cells and incubated for 24, 48 and 72 h with 3 repetitions for each ratio. Then, WST-1 test was applied. Cells grown in 96-well culture dishes were completely removed from the culture medium to perform WST-1 assay and incubated with 10% WST-1 reagent in the dark for 4 h. After this incubation period, a 540 nM measurement was performed on a 96-well culture dish spectrophotometer (Biotek, EL_X_800, SpectraLab, Lathamn, NY, USA) [14].

### 2.4. Apoptotic Staining

Tali™ Apoptosis Kit - Annexin V Alexa Fluor™ 488 & Propidium Iodide (Invitrogen™, ThermoFisfer, USA) was used for apoptosis determination. In this method, cells were stained using Annexin V AlexaFluor^®^ 488 and propidiumiodide. Apoptotic cells are green, dead cells are red, and live cells show little or no fluorescence. Application was made with the 48 h doses (BA (IC_50_), Cis (IC_50_) and BA (IC_50_)+Cis (IC_50_)) determined in the previous step and centrifugation was performed. Then, 200 µL of 1X Annexin binding buffer was added to the cells collected at the bottom of the tube. Next, 5 µL of Annexin V AlexaFluor^®^ 488 (Component A) was added to 100 µL of the mixture and kept in the dark for 20 min. Centrifugation was performed again and 100 µL of 1X Annexin binding buffer was added to the cells that settled at the bottom and vortexed. Then, 1 μL of Tali^®^ PropidiumIodide (PI, component B) solution was added to the final mixture and incubated in the dark for 5 min. Then, 25 μL of the mixture was taken and dropped onto special slides prepared for Tali and reading was performed with the analysis program. This was photographed using the Thermo EVOS^®^ FL ImagingSystem using brightfield mode and fluorescence mode, using a DAPI filter at 20x objective magnification.

### 2.5. Inflammatory Cytokines Levels

To determine the anti-inflammatory effect of BA (IC_50_), Cis (IC_50_) and BA (IC_50_)+Cis (IC_50_) in Y79 cells for 48 h, TNF-α (Invitrogen, Cat #13-7341-81), IL1-β (Invitrogen, Cat #PA1-84913) and IFN-γ (Invitrogen, Cat #14-7311-81) levels were determined in cell lysates using a multiplate reader device at 540 nm using specific commercial kits (ThermoFisher, USA ). The obtained data were divided by the total protein concentrations of the samples. Thus, normalized cytokine levels were expressed as ng/mg-protein or pg/mg-protein. 

### 2.6. Total RNA Isolation and cDNA Synthesis

In order to detect changes in gene expression of cultured cells as a result of BA (IC_50_), Cis (IC_50_) and BA (IC_50_)+Cis (IC_50_) treatment for 48 h, RNAs of cancer cells were purified with RNA isolation kit (ThermoFisher, USA). After isolation, RNA concentration was measured with a spectrophotometer and converted to cDNA with cDNA synthesis kit (Life Technologies, ThermoFisher, USA) as 1 μg total RNA. Amplification of target genes was performed with SYBR green dye in a LightCycler 480-II (ThermoFisher, USA ) real-time quantitative PCR device. Target gene and appropriately selected reference genes were amplified in the same panel. PCR conditions were applied as 10 min incubation at 95 °C, followed by 45 cycles of 15 s denaturation at 95 °C, 1 min annealing and extension at 60 °C. The Ct values of the peaks obtained during the amplification process were used to determine gene expressions and gene expressions were calculated with the 2^−∆∆Ct^ method. The primers used to investigate changes in the expression of these genes are given below in 5′-3′ order.

*NF-κB: F: GCG CAT CCA GAC CAA CAA TAA C, R: GCC GAA GCT GCA TGG ACA CT*
*p53: F: CACGAGCGCTGCTCAGATAGC, R: ACAGGCACAAACACGCACAAA*
*Caspase-3: F: GGTATTGAGACAGACAGTGG, R: CATGGGATCTGTTTCTTTGC*
*β-Actin: F: CCTCTGAACCCTAAGGCCAAC, R: TGCCACAGGATTCCATACCC*

### 2.7. Gene Ontologies (GO)

Three types of gene ontologies (GOs) were performed on possible target genes: cellular component, biological process and molecular function. The STRİNG program was used to evaluate these data. The rationale behind performing GO analysis in our study was to comprehensively characterize the biological processes, molecular functions and cellular components associated with the differentially expressed genes upon boswellic acid treatment in retinoblastoma cells. By conducting GO analysis, we aimed to identify key biological pathways, understand the functional relevance of differentially expressed genes and strengthen data interpretation. Instead of focusing solely on individual genes, GO analysis allows us to identify broader biological trends, helping to elucidate the molecular mechanisms underlying boswellic acid’s inhibitory effects, particularly in relation to *NF-κB* suppression. This approach provides an unbiased validation of our findings by linking gene expression changes to known tumor-suppressive or apoptotic pathways.

### 2.8. Statistical Analysis

The raw data compiled as a result of laboratory analyses were defined as mean ± standard deviation. SPSS 20.0 software was preferred for statistical evaluations. In statistical evaluation, whether there was a statistical difference in parameters showing normal distribution was determined with the ANOVA test. Whether there was a statistical difference between groups in parameters not showing normal distribution was determined with the Kruskal–Wallis test. The groups between which the difference occurred were determined with the Mann–Whitney U test. Expression levels of genes showing statistical differences were indicated with (*) in the graphs.

## 3. Results

### 3.1. WST-1 Analysis Findings

**Figure 1:**
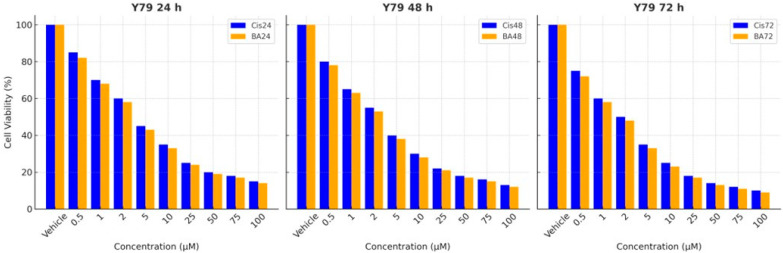
Boswellic acid and Cisplatin cytotoxicity in Y79 cells. BA IC_50_ and Cis IC_50_ were determined after 24, 48 and 72 h of BA and Cis application. The figure shows cell viability (%) of Y79 cells treated with Cis and BA at different concentrations (0.5–100 μM) for 24 h, 48 h and 72 h. The bar graphs compare the effects of Cis (blue) and BA (orange) over time. Cell viability decreases in a dose- and time-dependent manner for both treatments. IC_50_ values indicate that Cisplatin is slightly more effective than BA, with lower IC_50_ values at 48 h (Cis: 2.18, BA: 2.06) and 72 h (Cis: 1.48, BA: 1.84). At higher concentrations (≥10 μM), both treatments significantly reduce cell viability.

To determine the cytotoxicity of boswellic acid and Cisplatin in Y79 cells, eight different doses ranging from 0.5 to 100 μM were used. It was seen that the cytotoxicity of BA and Cis increased at doses of 0.5 μM and higher. It was determined that they might have high cytotoxicity at doses of 2 μM and above. Figure 1, prepared based on WST-1 analysis data, shows that BA and Cis decreased cell viability in a dose-dependent manner. The obtained data were evaluated by probit analysis, and the acute mean toxic dose (IC_50_) of BA in Y79 cells was determined as 2.82 μM after 24 h exposure, as 2.06 μM for 48 h and as 1.84 μM for 72 h. The IC_50_ dose of Cis was determined as 2.96 μM for 24 h, 2.16 μM for 48 h and 1.48 μM for 72 h (Figure 1).

These results suggest that both Cisplatin and boswellic acid exhibit cytotoxic effects on Y79 cells, with increasing potency over time.

### 3.2. Inflammatory Cytokines Levels Findings

**Figure 2:**
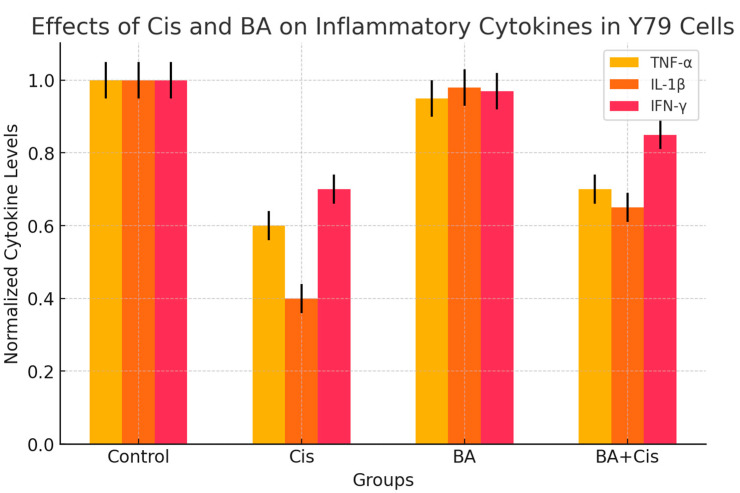
Effects of BA (IC_50_), Cis (IC_50_) and BA (IC_50_) + Cis (IC_50_) on inflammation in Y79 cells for 48 h. Inflammation markers TNF-α, IL1-β and IFN-γ were measured using ELISA kits. IL1-β and IFN-γ were determined as pg/mg and TNF- α as ng/mg. The bar graph represents the effects of Cis and BA on inflammatory cytokine levels (TNF-α, IL1-β, and IFN-γ) in Y79 cells. Cis treatment significantly decreased TNF-α, IL1-β and IFN-γ levels compared to the control group, with the greatest reduction observed in IL1-β. BA treatment did not significantly alter cytokine levels compared to the control group. BA + Cis treatment significantly reduced TNF-α and IL1-β levels (*p* < 0.05), but the decrease in IFN-γ was not statistically significant (*p* > 0.05).

When the effects of Cis on inflammation in Y79 cells were examined, it was seen that the changes in TNF-α, IL1-β and IFN-γ levels were significant when compared to the control group. The highest decrease occurred in IL1-β in the Cis group. When the BA group was compared with the control group, there was no significant difference in terms of the effect on inflammation in the cells. When the control group was compared with the BA+Cis group, the decrease in TNF-α and IL1-β levels was significant (*p* < 0.05), while the decrease in IFN-γ was not significant (*p* > 0.05) (Figure 2).

### 3.3. Annexin V Apoptotic Staining Findings

**Figure 3:**
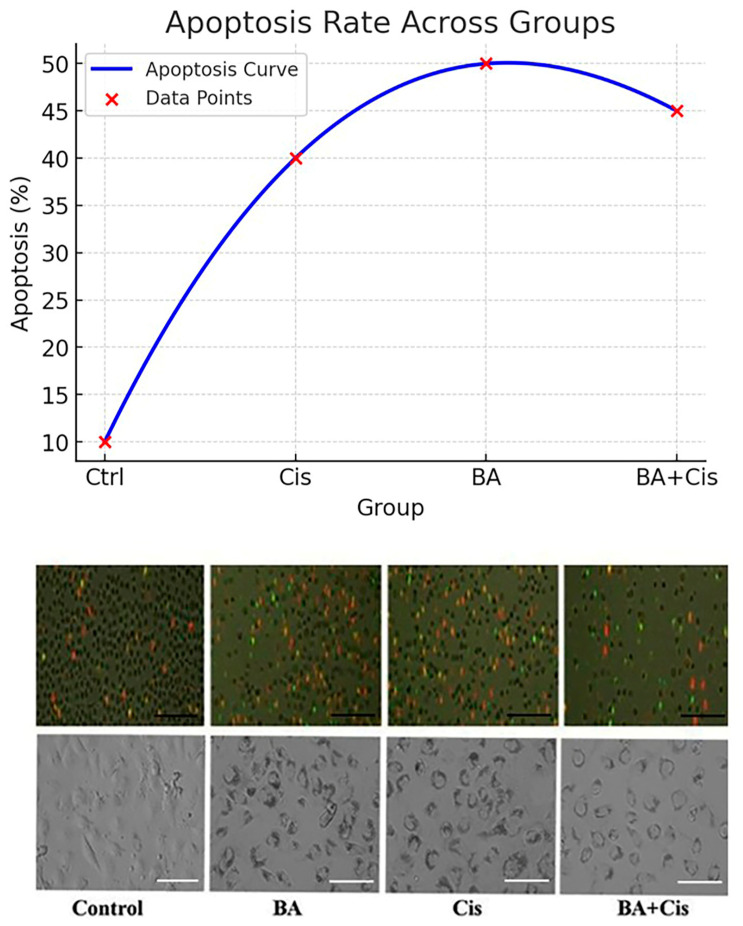
BA and Cis IC50 for 48 h proportions of live, dead, and apoptotic cells by Annexin V apoptotic staining (magnification: X20, scale bar: 50 μ). The figure presents apoptosis analysis across different treatment groups: Control (Ctrl), Cis, BA, and BA + Cis. The top panel displays an apoptosis trend curve, illustrating a polynomial fit based on the apoptotic percentages observed in each group. The curve shows a significant increase in apoptosis in the treatment groups (Cis, BA, and BA + Cis) compared to the control, with BA inducing the highest apoptosis, followed by BA + Cis. The bottom panel contains fluorescent and phase-contrast microscopy images. Fluorescence staining reveals apoptotic cells (red) and live cells (green), with a greater proportion of apoptotic cells in the treated groups. The phase-contrast images show apoptotic morphological features, including cell shrinkage and membrane blebbing.

The findings show that BA can increase the therapeutic effect when combined with chemotherapy drugs used in many cancer treatments. It has been shown that BA can exhibit a synergistic effect with Cis and can also play a preventive role against Cis toxicity (Figure 3).

Overall, the results suggest that boswellic acid enhances apoptosis in retinoblastoma cells, both alone and in combination with Cisplatin, supporting its potential as an anticancer agent.

### 3.4. RT-PCR Findings

**Figure 4:**
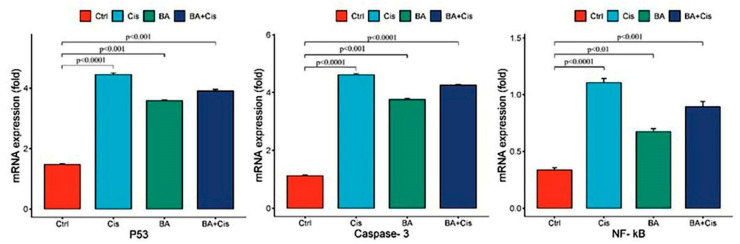
Relative fold increase values of *p53* and *Caspase-3* gene expressions in Y79 cell lines and decrease value of *NF-κB* expressions, IC_50_ doses for 48 h after single and combined drug administration. The figure presents the mRNA expression levels of *p53*, *Caspase-3* and *NF-κB* under different treatment conditions: Control (Ctrl), Cis, BA and BA + Cis. The *y*-axis represents the fold change in mRNA expression, while the *x*-axis indicates the treatment groups. Statistical significance is indicated by *p*-values, with notable differences between groups. For *p53* and *Caspase-3*, mRNA expression is significantly increased in the Cis group compared to the Ctrl group (*p* < 0.0001). The BA group also shows elevated expression, but the highest increase is observed in the BA + Cis combination group. For *NF-κB*, mRNA expression is also significantly upregulated in the Cis group compared to the Ctrl (*p* < 0.0001). However, the BA group exhibits a lower *NF-κB* expression compared to Cis, and the BA + Cis group shows an intermediate level.

Treatment of Y79 cells with BA and Cis resulted in an increase in *NF-κB*, *p53* and *Caspase-3* gene expression. The effects of BA and Cis on apoptosis (*p53* and *Caspase-3*) and proliferation (*NF-κB*) in Y79 cells were determined by comparing the mRNA expression levels of each group of analyzed genes with the control group. When BA+Cis was applied to Y79 cells at an IC_50_ dose for 48 h, it was seen that apoptotic *p53* and *Caspase-3* gene expressions were stimulated at a statistically significant level compared to the control group (Figure 4). When BA+Cis was applied to Y79 cells at an IC_50_ dose for 48 h, it was determined that the expression levels of *NF-κB* gene showed statistically significant differences compared to the control group (Figure 4). Among the apoptotic genes, the highest effect of *p53* and *Caspase-3* was observed only in the Cis-applied group, while the highest effect of *NF-κB* was observed in the Cis-applied group.

Overall, these results suggest that boswellic acid enhances the apoptotic markers (*p53* and *Caspase-3*) while modulating *NF-κB* expression, potentially contributing to its anticancer effects when combined with Cisplatin.

### 3.5. Enrichment Analysis

**Figure 5:**
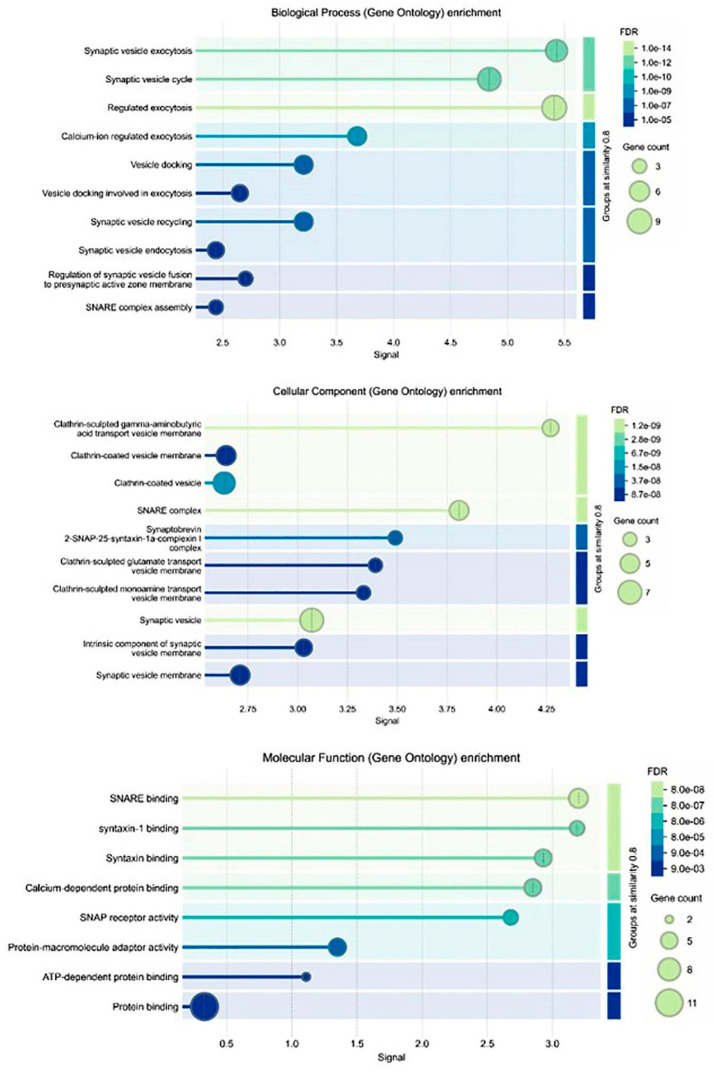
Gene ontologies (biological process, cellular component and molecular function) analysis. The figure presents a three-panel Gene Ontology enrichment analysis covering Biological Process, Cellular Component and Molecular Function. Each panel shows significantly enriched GO terms (*y*-axis) plotted against a signal value (*x*-axis). Bubble size indicates the number of genes associated with each term, while bubble color reflects the level of statistical significance (False Discovery Rate). In the Biological Process panel, terms related to synaptic vesicle exocytosis and regulated exocytosis are highly enriched. The Cellular Component panel highlights structures such as clathrin-coated vesicles and synaptic vesicles, while the Molecular Function panel features enrichment in soluble NSF attachment protein receptor binding and syntaxin binding. A similarity threshold of 0.8 is used to group related terms, as shown by the shaded area on the right side of each plot.

**Figure 6:**
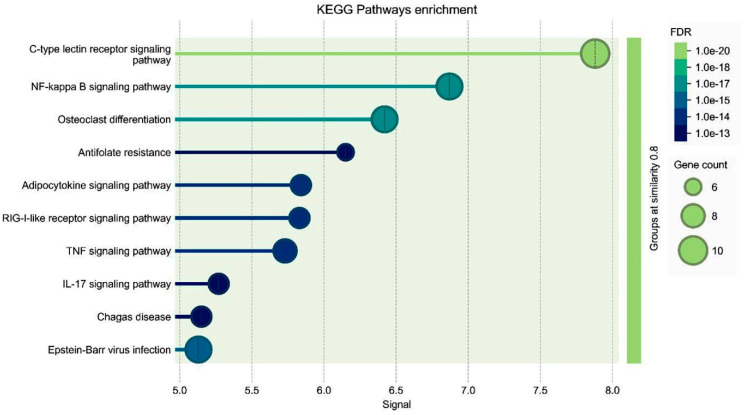
Enrichment analysis for the 120 common compound targets for *NF-κB* signaling pathway. The figure presents KEGG Pathways enrichment analysis, visualizing the significantly enriched pathways associated with the gene set. The *y*-axis lists the identified pathways, including C-type lectin receptor signaling pathway, *NF-κB* signaling pathway, osteoclast differentiation, antifolate resistance, adipocytokine signaling pathway, retinoic acid-inducible gene I-like receptor signaling pathway, TNF signaling pathway, IL-17 signaling pathway, Chagas disease, and Epstein–Barr virus infection. The *x*-axis represents the signal values, indicating the degree of enrichment. Each pathway is represented by a bubble, where bubble size denotes the gene count contributing to each pathway, with larger bubbles representing a higher number of genes. Bubble color represents the False Discovery Rate, with darker blue indicating higher statistical significance and lighter green representing lower significance. A grouping similarity threshold of 0.8 is applied, as shown by the shaded green region on the right side of the figure. The most enriched pathways include the C-type lectin receptor signaling pathway, *NF-κB* signaling pathway and osteoclast differentiation, which have the highest signal values and gene counts.

Analysis findings show only important functions (Figure 5 and Figure 6). Target genes were found to be involved in various cellular components in the biological process category, such as I-κB kinase/*NF-κB* signaling, *NF-κB*-inducing kinase signaling, etc. (Figure 6). In terms of cellular components, there was the NF-κB complex, I-κB/*NF-κB* complex, intracellular protein-containing complex. It was found that the molecular function category exhibited roles such as transferrin receptor binding, I-κB kinase activity, identical protein binding (Figure 5). KEGG pathway enrichment analysis of target genes was performed with STRING program. The findings showed that 120 genes were involved in the enrichment process and 90 pathways were apoptosis related, exhibiting a significant correlation with target genes (*p* < 0.05). Basically, C-type lectin receptor signaling pathway, *NF-κB* signaling pathway and TNF signaling pathway are shown (Figure 6).

## 4. Discussion

The results of this study demonstrate that BA, in combination with Cis, effectively inhibits the growth of Y79 retinoblastoma cells by inducing apoptosis and modulating *NF-κB* signaling. WST-1 analysis revealed that both BA and Cis exhibited dose- and time-dependent cytotoxic effects on Y79 cells, with IC_50_ values decreasing over time. The BA + Cis combination showed the strongest inhibitory effect on cell viability, suggesting a potential synergistic interaction between the two agents. Our findings further show that BA enhances the expression of apoptotic markers *p53* and *Caspase-3*, supporting its pro-apoptotic role in retinoblastoma cells. However, *NF-κB* mRNA expression was upregulated in all treatment groups, with the highest levels observed in the Cis-treated group, while BA treatment alone exhibited relatively lower *NF-κB* expression. This suggests that although BA does not directly suppress *NF-κB* transcription, it may modulate its functional activity through post-translational mechanisms such as phosphorylation, nuclear translocation, or interaction with inhibitory proteins. The BA + Cis combination resulted in an intermediate *NF-κB* expression level, implying a potential regulatory effect rather than direct inhibition. Given the complexity of *NF-κB* signaling, further research is required to investigate *NF-κB* protein levels, phosphorylation status, and nuclear translocation to determine the exact mechanism by which BA influences this pathway in retinoblastoma cells.

New drugs are being developed by utilizing natural resources to treat many diseases. Although it is more expensive and laborious to discover and develop new therapeutic agents in natural resources, it provides an abundant source for new drugs. Natural resources continue to be a rich and abundant source of potential new drugs. One of these sources is boswellic acid. BA has very strong biological and pharmacological potentials and contains pentacyclic triterpenoids that attract the attention of synthetic and medicinal chemistry. It shows that natural BA and its synthetic derivatives can play an important role in the treatment of many inflammatory diseases, especially cancer. Moreover, BA have been used in the treatment of various diseases in formulations with active compounds [15]. Particular combination ideas are extremely important in reducing toxicity and resistance. Moreover, the interactions of BA with other anticancer drugs should be fully investigated, because they are expected to increase synergism with anticancer and anti-inflammatory effects. The most important challenge in drug discovery is to eliminate drug resistance and to ensure the development of molecules with a new target. Therefore, we investigated the effects of the combination of BA with a potent anticancer agent Cis and the obtained results confirm the synergism. While apoptotic gene expressions increased, an increase in anti-inflammatory biochemical parameters was also observed. In other words, while killing cancer cells, it creates a microenvironment that will allow healthy cells to survive.

In vitro studies on different cancer cell lines have highlighted the strong anticancer properties of BA. It has been reported that BA treatment in the colon cancer cell line HCT-116 caused a decrease in cyclin-dependent kinases such as cyclin D, cyclin E, CDK2 and CDK4 [16]. In another study, Acetyl-beta boswellic acid and BA stopped the activity of I-κB kinase in chemotherapeutic androgen-independent PC-3 prostate cancer cells in vitro and in vivo, inhibiting activated *NF-κB* signaling, reducing proliferation and leading to cell death [17]. It has been shown that apoptosis induced by TNF and chemotherapeutic agents is more effective and suppresses TNF-dependent invasion. In pancreatic cancer cell lines, BA treatment has been shown to inhibit the expression of *NF-κB*, and thus suppress COX-2 and MMP-9 genes. In addition, in an experimental adenomatous polyposis model in which BA and aspirin were applied, it was shown that BA caused regression in adenomatous polyps by modulating the Wnt/β-catenin pathway and the *NF-κB*/COX-2 pathway in mice [18]. It was found that inhibition of *NF-κB* with an extract containing Boswellia decreased the expressions of VEGF, TNF-α and MCP-1 and improved mouse liver granuloma [19]. It was shown that apoptosis was induced and tumor regression was triggered by the reduction in *NF-κB* in cancers treated with 3-α-Butyryloxy-β-boswellic acid, a semi-synthetic analog of BA [20]. Colorectal cancer is one of the most common cancers. In HT-29 human colon cancer cells, Boswellia serrata (B. serrata) has anticancer activity. A significant difference was observed in B. serrata-treated HT-29 compared to the control group [21].

In the presented study, it was determined that oxidative stress and, accordingly, apoptotic genes *p53* and *Caspase-3* mRNA expression levels were stimulated due to the increase in IL1-β and IFN-gamma levels in the group to which BA was applied at a 48 h IC_50_ dose (Figure 4). Indeed, studies have indicated that cytosolic and mitochondrial reactive oxygen species, which can also play an active role in *p53* stimulation, can be effective in the induction of apoptosis [22]. However, a difference was observed in the experimental group to which BA+Cis was applied compared to the control group in terms of oxidative stress and inflammation, and apoptosis was observed to be stimulated. In addition, the presented study is the first study in which natural antioxidant and anticancer agent and a strong chemotherapy agent Cis were applied together. In previous studies, it has been reported that natural sources such as gallic acid and rosmarinic acid exhibit effective anticancer interactions with doxorubicin [23,24]. When we compare the reports of these studies with our results, we see that combination therapy can eliminate secondary effects in many cancer treatments. In addition, cytotoxicity and results that can directly affect survival are seen.

While this study provides valuable insights into the potential therapeutic effects of BA in combination with Cis on Y79 retinoblastoma cells, it has several limitations. First, *NF-κB* activity was assessed only at the mRNA level, and functional validation through protein expression, phosphorylation status, and nuclear translocation assays was not performed. Future studies should include Western blot and immunofluorescence analysis to confirm whether *NF-κB* activation is modulated post-translationally. Second, the study was conducted in vitro, and while the results suggest a promising anticancer effect, in vivo models are necessary to evaluate the pharmacokinetics, bioavailability, and systemic effects of BA in retinoblastoma treatment. Lastly, potential off-target effects and interactions with other cellular pathways remain unexplored, requiring further mechanistic studies to fully understand BA’s role in *NF-κB* signaling and apoptosis regulation.

Future research should focus on elucidating the precise molecular mechanisms by which BA modulates *NF-κB* signaling, particularly at the protein level. Since our study demonstrated *NF-κB* mRNA upregulation but lacked functional validation, subsequent investigations should employ Western blot analysis, phosphorylation assays and nuclear translocation studies to determine whether BA affects *NF-κB* activation post-translationally. Additionally, in vivo studies are crucial to evaluate the bioavailability, pharmacokinetics and systemic efficacy of BA in retinoblastoma models. Further research should also explore potential synergistic or antagonistic effects of BA with other chemotherapeutic agents to optimize combination therapies. Finally, given *NF-κB*’s dual role in inflammation and apoptosis, future studies should investigate how BA influences *NF-κB*-dependent gene expression networks and downstream pathways, ensuring a comprehensive understanding of its therapeutic potential in retinoblastoma treatment.

## 5. Conclusions

This study demonstrates that BA enhances apoptosis and modulates *NF-κB* signaling in Y79 retinoblastoma cells, particularly when combined with Cis. The combination of BA + Cis exhibited the strongest cytotoxic effect, suggesting a potential synergistic interaction. Our findings indicate that BA increases *p53* and *Caspase-3* expression, while *NF-κB* mRNA expression was upregulated, particularly in the Cis-treated group. However, this does not necessarily imply increased functional *NF-κB* activity, as post-translational regulatory mechanisms may play a role.

## Data Availability

All details about the study can be obtained from the corresponding author.

## Abbreviations

Boswellic acid	BA
Cisplatin	Cis
Human retinoblastoma cell line	Y79
Water soluble tetrazolium-1	WST-1
Interleukin 1-beta	IL1-β
Tumor necrosis factor-α	TNF-α
Interferon γ	IFN-γ
Protein53	p53
Nuclear Factor Kappa B	NF-κB
Gene ontologies	GO
